# Effect of Dry-Period Diet on the Performance and Metabolism of Dairy Cows in Early Lactation

**DOI:** 10.3390/ani10050803

**Published:** 2020-05-06

**Authors:** Julien Soulat, Emilie Knapp, Nassim Moula, Jean-Luc Hornick, Céline Purnelle, Isabelle Dufrasne

**Affiliations:** 1 Department of Veterinary Management of Animal Ressources, Faculty of Veterinary Medicine, Fundamental and Applied Research for Animal & Health (FARAH), University of Liège, 4000 Liège, Belgium; julien.soulat@live.fr (J.S.); emilie.knapp@quartes.com (E.K.); nassim.moula@uliege.be (N.M.); jlhornick@uliege.be (J.-L.H.); 2 Institut Supérieur Industriel Agronomique, Haute Ecole Charlemagne, 4500 Huy, Belgium; celinepurnelle@gmail.com; 3 Centre des Technologies Agronomiques (CTA), 4577 Strée, Modave, Belgium

**Keywords:** dry period, fatty acids, milk yield, serum metabolites

## Abstract

**Simple Summary:**

In dairy cows, the management of the dry period should optimize milk production and limit impact on metabolic health. In early lactation, there is little information on the effect of the dry-period diet composition on the production and composition of milk and the blood metabolites. The aim of this study was to compare the effects of different dry-period diets on the blood metabolites of dairy cows during the peripartum period and the milk yield and fatty acid profile at onset of lactation. This study showed that different dry-period diets can be used without impact on milk production and composition when these diets cover the needs of dairy cows. However, blood metabolites were more sensitive to the diet offered during the dry period. In early lactation, a dry-period diet based on corn and grass silages allowed a smooth transition with better rumen and liver function parameters.

**Abstract:**

The objective of this work was to observe the effect of three different dry-period diets on blood metabolites (*p* = 9) and the production and fatty acid (FA) profile of milk (*p* = 19) in the peripartum period. In this study, 32 Holstein dairy cows, during their dry period, were divided in 3 different diet groups, as follows: the CONC diet (n = 11) was based on concentrate meal and straw, the CORN diet (n = 11) was based on corn silage, and the MIXED diet (n = 10) was based on corn and grass silages. According to our results, the variations of C18:2n-6, C18:3n-3, non-esterified fatty acid (NEFA), NEFA/cholesterol ratio, and albumin were significantly (*p* < 0.05) different, according to the dry diet. The dry-period diet also had a significant effect on the concentrations of urea and vitamin B_12_ in the blood. In early lactation, this work showed that blood metabolites were more sensitive to changes in the dry diet than the production and FA profile of milk.

## 1. Introduction

The dry period plays a key role in preparations for calving as well as the milk production and health of dairy cows [1]. Adequate management of this period optimizes the development of the mammary gland and fetus in the last month of pregnancy, as well as the milk production of the next lactation, and also has an impact on cows’ metabolic health and reproductive performance [1]. During this period, dairy cows restore their body reserves, and repair and regenerate the alveolar system in the mammary gland [2] to anticipate the needs of the next lactation. During the peripartum period (i.e., three weeks before and three weeks after calving), cows have a high energy demand and must adapt to lactation demands (milk and colostrum productions), with a decrease in dry matter intake in the last month of pregnancy. In early lactation, dairy cows have a negative energy balance (NEB) [3]. The NEB stimulates fat mobilization to compensate for the limited feed intake in the form of non-esterified fatty acids (NEFA) and, in some cases, subsequent accumulation of β-hydroxybutyrate (BHB) in the blood [4]. The plasma concentrations of cholesterol, glucose, BHB, and NEFA, and the plasma NEFA/cholesterol ratio provide information on the metabolic status of the cows [5,6]. In the serum, the total proteins and albumin are indicators of the inflammation state, associated with fat mobilization [7]. Concentrations of urea and vitamin B_12_ in the blood give information on rumen performance [8,9]. The intensity of the NEB decreases milk production [10]. In early lactation, due to the increase in energetic needs, hepatic gluconeogenesis is stimulated to increase glucose supply. Lipolysis is favored and the NEFA content in the blood rises; these NEFA are used for milk fat synthesis or as an energy source [11]. A high body condition score (BCS) causes lipolysis more important around calving with the increase in NEFA concentrations in the blood [3]. Many studies reported the impact of the energy source (lipogenic vs. glycogenic) and/or energy level in the dry-period diet on the production and composition of milk [6], blood metabolite concentrations [12], and the health of dairy cows [13]. The supplementation of proteins in the dry diet can increase milk yield [14], limit BCS loss [15], and reduce health problems [16]. This supplementation can also induce an important NEB in early lactation [17]. Other studies did not observe an effect of the diet during the dry period on the NEB [18,19]. However, there is little information on the effect of the dry-period diet composition on the production and composition of milk, the blood metabolites, and BCS in early lactation. The objective of the current study was to compare the effects of three dry-period diets, commonly used in commercial farms, differing in terms of starch, fiber and protein, on the blood metabolites of dairy cows during the peripartum period and milk composition and yield at onset of lactation. The study also tested the hypothesis that milk fatty acid composition is related to changes in blood, in the early lactation of dairy cows.

## 2. Materials and Methods

### 2.1. Animals, Experimental Design, Diets, and Housing

The Animal Ethics Committee of Liège University (Liège, Belgium) approved the experimental protocol (No. 1942), in compliance with the guidelines established by the European Union Directive 2010/63/EU. The experiment was conducted at the Centre des Technologies Agronomiques (Strée, Modave, Belgium) between 21 August 2017 and 1 August 2018. Thirty-two Holstein dairy cows (24 multiparous and 8 primiparous) participated in the trial from their dry period up to 100 days in milk (DIM). According to the calving calendar, the 32 dry cows were allocated to one of three dietary treatment groups, which were balanced according to parity and projected milk production for the multiparous. The dry cows were housed in a free stall alone or in a group (a maximum of 4 cows) without access to pasture. They received intramammary treatment with a long-acting antimicrobial and nonantibiotic internal teat sealant, for mastitis prevention. The forage and concentrates used in each dietary treatment were the same throughout the experiment. The components of the diets were weighed every day, except straw, which was weighed three times per week.

The dietary treatments were as follows: treatment 1 (*n* = 11, calving from 4 October to 10 December 2017), a dry-period diet based on concentrate meal (Lactodry, Quartes, Deinze, Belgium) and straw (CONC); treatment 2 (*n* = 11, calving from 18 December 2017 to 23 April 2018), a diet based on corn silage (CORN) and concentrate (Synchromix P2, Quartes, Deinze, Belgium); and treatment 3 (*n* = 10, calving from 10 December 2017 to 29 March 2018), a diet based on corn and grass silages (MIXED). The composition and nutritional values of the dietary treatments given during the first weeks of the dry period are presented in Table 1.

During the weeks preceding calving, the cows received a supplement of concentrate. More precisely, in the CONC diet, during weeks −3 and −2 relative to the parturition of the cows, the quantity of Lactodry (Quartes, Deinze, Belgium) increased by 1 kg/day. A further increase of 1 kg/day was put in place for the week before calving. For the other groups (i.e., CORN and MIXED), 1.5 kg/day of concentrate (Nutritop, Quartes, Deinze, Belgium) was provided for weeks −2 and −1 before calving. The chemical composition of the Nutritop (g/kg of dry matter (DM)) was as follows: DM = 881; crude protein (CP) = 205; starch = 300; sugar = 41; crude fiber = 58; crude fat = 41; intestinal digestible protein (DVE) = 114; degraded protein balance (OEB) = 31; and net energy (NE) = 8.0 MJ/ kg of DM. In order to prevent hypocalcemia, the multiparous cows received an injection of vitamin D3 (500,000 IU per cow) just before calving. A solution composed of calcium and phosphor salts (59 and 45 g, respectively, per cow) were also given within the 4 h around the calving, to each cow.

After calving, the cows received the same base lactation diet described in Table 1. They also received two concentrates: Topmilk (Quartes, Deinze, Belgium) and a mixed concentrate (45% of Lactospeed, 36% of Lacto Pature and 18% of Lactopro 40, Quartes, Deinze, Belgium), by a computerized feeder, in a freestall. The dairy cows received 1.5 kg/day of Topmilk until the 70th DIM. The chemical composition of the Topmilk (g/kg of DM) was as follows: DM, 880; CP, 199; sugar, 36; DVE, 119; OEB, 22; NE, 8.6 MJ/kg of DM. According to their milk production, the dairy cows received between 1 and 5.5 kg/day of the mixed concentrate. The chemical composition of the mixed concentrate (g/kg of DM) was as follows: DM, 871; CP, 256; sugar, 56.5; DVE, 163; OEB, 36; NE, 7.8 MJ/kg of DM. During the dry and lactation periods, the diets were served twice a day (07:00 and 17:00 h). Throughout the duration of the study, the dairy cows had free access to water and the base diet (dry or lactation diet). Net energy was calculated using the Dutch NE system for lactation (VEM) where 1000 VEM = 6.9 MJ of NE [21]. Nutritional values of the concentrates used were obtained from the manufacturer (Quartes, Deinze, Belgium) and the forages were analyzed by near-infrared spectrometry (Laboratoire de la Province de Liège, Belgium).

The BCS of cows were assessed by the same trained person, once a week at different periods (weeks −1, +1, +2 and +3 relative to parturition), using the visual technique on a scale of 1 (severe under conditioning) to 5 (severe over conditioning) with 0.25 point intervals [22]. Information on the number of diseases diagnosed and treated (e.g., milk fever, clinical mastitis, lameness) was collected for each cow during the whole experimental period.

### 2.2. Milk Samples and Analyses

The cows were milked twice a day at 06:00 and 16:00 h, respectively, in a double 3-parallel milking parlor with automatic milk yield registration (Delaval, Gent, Belgium). For each cow, the milk yield per day after calving to 100 days in milk (DIM) was considered.

Milk samples (30 mL) used for FA analysis were collected from two consecutive milkings (i.e., 50% morning and 50% evening) on the same day as blood sampling once per week, in weeks +1, +2 and +3 relative to parturition, and bronopol was added. These samples were stored at −20 °C until the end of the experimental period before being sent off for analysis. Milk analyses were performed at Comité du Lait (a certified milk control station, Battice, Belgium; Belgian accreditation number 262-TEST in compliance with ISO 17025) by mid-infrared spectroscopy, using a MilkoScan FT6000 spectrophotometer (Foss Electric, Hillerød, Denmark). The FA predictions were performed using the models developed by Soyeurt et al. [23] and updated by Grelet et al. [24].

### 2.3. Blood Samples and Analyses

Blood samples were collected after the morning milking, between 09:00 and 10:00 h during the morning feeding. Blood (8 mL per tube) was collected from the coccygeal vein into two evacuated and uncoated (BD Vacutainer SST II Advance, Plymouth, UK) tubes and two heparin-coated (BD Vacutainer LH 170 I.U., Plymouth, UK) tubes. Blood samples were collected once per week at weeks −1, +1, +2 and +3 relative to parturition. After each collection, one uncoated and one heparin-coated tube were sent to the laboratory (Synlab Veterinary, Liège, Belgium).

Directly after blood sampling, the second heparin-coated tube was used to evaluate glucose and BHB concentrations, using an electronic handheld device (FreeStyle Optium H, Abott GmbH & Co. KG, Wiesbaden, Germany). This test system used an electronic handheld meter and a specific electronical test strip to measure the glucose (FreeStyle Optium H Blood glucose test strips, Abbott Diabetes Care, Witney, UK) and BHB (FreeStyle Optium H β-Ketone, Abbott Diabetes Care, Witney, UK). The second uncoated tube was kept in the freezer. Serum NEFA (photometric measurement method; Randox NEFA, Crumlin, UK), vitamin B_12_ (chemiluminescent microparticle intrinsic factor assay method; ARCHITECT B12 7K61, Abbott, Longford, Ireland), cholesterol (enzymatic coloration test; Cholesterol OSR6116), urea (ultraviolet kinetic test; Urea OSR6134), albumin, and total serum proteins (photometric color test; Albumin OSR6102 and Total protein OSR6132, respectively; Beckman Coulter, Krefeld, Germany) were measured in a commercial laboratory (Synlab Veterinary, Liège, Belgium) with a clinical chemistry analyzer (Beckman Coulter, Krefeld, Germany).

### 2.4. Statistical Analyses

The statistical analyses were performed using R 3.5.1 software [25]. First, descriptive analysis of the data, using a quantile–quantile plot, was performed to observe the normality of each variable [26]. As all milk and blood measures were repeated in time, an ANOVA with repeated values (mixed model) was performed for each parameter. An ANOVA was used to evaluate their dependence on the diet intake during the dry period, the period, and the interaction between the diet and the period. Each mixed model included three fixed effects: period, dietary treatment during the dry period and dietary treatment × period interaction, and the random effect of the individual animal. If the interaction in the mixed model performed was above *p* > 0.10, a new mixed model was performed without the interaction. A post hoc analysis of each mixed model was graphically performed with quantile–quantile plots on the fixed effects and on the random effect [26]. Mixed models were performed using the package “lmerTest” [27].

ANOVA (without random effect) were conducted for the period duration, the number of lactations, the peak of lactation, and the milk yield parameters, to observe the effect of the three dietary treatments.

If the results of the mixed models and ANOVAs were significant (*p* ≤ 0.05), a Tukey test was performed to compare the average pairwise, using the “emmeans” [28] and “multcompView” [29] packages for mixed models and “agricolae” package [30] for ANOVAs. *p* > 0.05 and ≤0.10 were considered as trends.

According to the milk or blood traits considered in this study, the time step considered was the week.

Pearson’s correlations were performed between the different FA and blood metabolites at weeks +1, +2 and +3 relative to parturition.

To analyze the disease data, a chi-squared test was conducted for each disease to observe the relationship between the dietary treatment and the number of sick cows.

## 3. Results

In the present study, the dry period’s duration and the parity of the dairy cows were the same between the three dietary treatments (Table 2). In early lactation (i.e., < 30 DIM), five dairy cows were treated for mastitis (CONC = 1, CORN = 2, and MIXED = 2) and one cow from MIXED treatment was treated for a milk fever. The effects of the dietary treatments were non-significant on mastitis (*p* = 0.53) and milk fever (*p* = 0.41).

### 3.1. Effect of the Diet during the Dry Period on Milk Production and Composition

**Figure 1:**
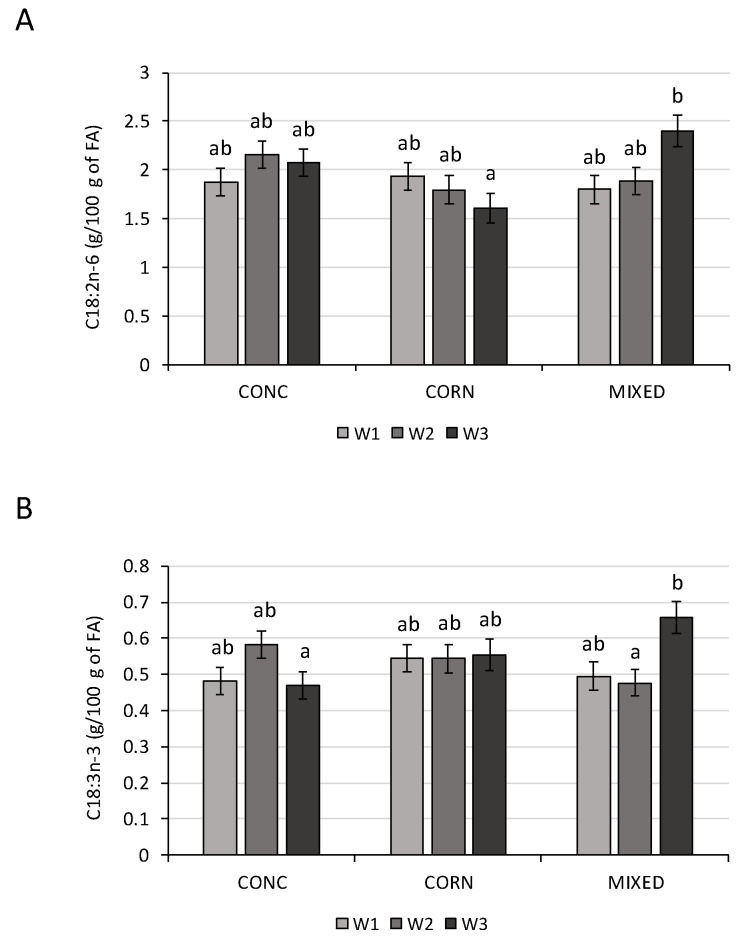
Variation of both the fatty acids (FA): C18/:2n-6 (**A**) and C18:3n-3 (**B**), between weeks +1 and +3 relative to parturition (W1 to W3), in cows with different dietary treatments (diet based on concentrate meal and straw (CONC), on corn silage (CORN) or corn and grass silages (MIXED)) given during the dry period and receiving the same lactation diet. Values followed by different letters (a, b) are significantly different (*p* ≤ 0.05).

The three diets fed during the dry period had few effects on milk production and FA profile during the three weeks (W1 to W3) after calving. During this lactation period, the milk yield was only affected by the period (Table 3). During the whole lactation period considered in this study (i.e., 100 DIM), the milk yield was only affected by the period (*p* < 0.001). As observed during the first weeks of lactation, the treatment during the dry period was not significant (*p* = 0.80). The quantity of milk produced at the peak of lactation was non-significantly different among the three dietary treatments (Table 2). Only the quantity of C18:2n-6 and C18:3n-3 was significantly affected by the interaction between the treatment and the period (Table 3). For the other FA and the milk yield, this interaction was not significant (*p* > 0.05). During the first and second week (W1 and W2) after calving, the quantities of C18:2n-6 and C18:3n-3 were not significantly different between the three-dietary treatments (Figure 1). However, at +3 weeks relative to parturition, the quantity of C18:2n-6 was significantly higher (*p* < 0.05) with the MIXED treatment than with the CORN treatment, and the quantity of C18:3n-3 was significantly higher with the MIXED treatment than the CONC treatment. According to the treatment, a tendency was observed for the variation quantity of C17:0 (Table 3).

### 3.2. Effect of the Diet during the Dry Period on the BCS of the Dairy Cows

In the present study, the dietary treatments tended to affect the BCS of the dairy cows (Table 4). The dairy cows from the MIXED treatment had a higher BCS than those from the CONC treatment. Just the period influenced significantly the BCS. The BCS of the dairy cows decreased significantly after parturition and continued until the week +3 relative to parturition. The effect of the dietary treatment x period interaction on the BCS was non-significant (*p* > 0.05).

### 3.3. Effect of the Diet during the Dry Period on the Blood Metabolites

**Figure 2:**
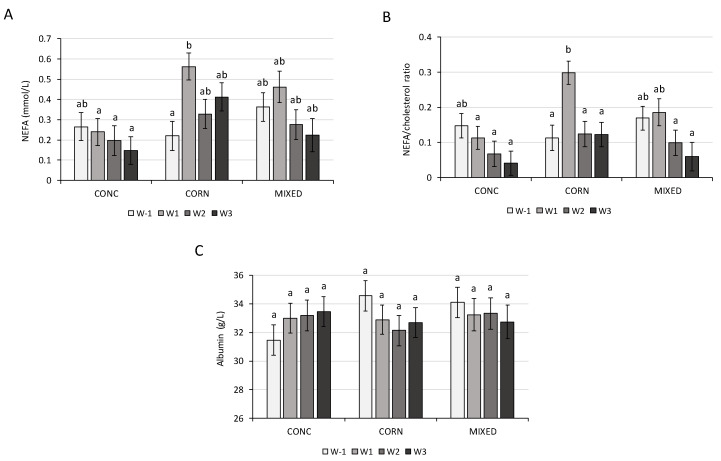
Variation of the blood metabolites: non-esterified fatty acids (NEFA, **A**); NEFA/cholesterol ratio (**B**) and albumin (**C**), between weeks −1 and +3 relative to parturition (W−1 to W3), in cows with different dietary treatments (diet based on concentrate meal and straw (CONC), on corn silage (CORN) or corn and grass silages (MIXED)) given during the dry period and receiving the same lactation diet. Values followed by different letters (a, b) are significantly different (*p* ≤ 0.05).

The results of the present study indicated that the dietary treatments affected the concentration of some metabolites (Table 4). The variations in NEFA, NEFA/cholesterol ratio, and albumin were significantly affected by the dietary treatment during the period considered (weeks −1 to +3 relative to parturition). At the second week relative to parturition, NEFA and the NEFA/cholesterol ratio were significantly higher in the CORN treatment than in the CONC treatment (Figure 2). At the other periods, these metabolites were not significantly different according to the dietary treatment. 

The serum albumin variation was different according to the dietary treatment. During the period considered, the albumin values increased with the CONC treatment and decreased with the other treatments. However, the Tukey analysis did not highlight significant differences. The dietary treatment also had a significant effect on the concentrations of serum urea and vitamin B_12_ (Table 4). The urea concentration was significantly higher with the CONC treatment than with the MIXED treatment, whereas for the vitamin B_12_ concentration, it was the opposite. These metabolites also varied significantly over time. The serum urea was significantly higher at weeks +1 and +3 relative to parturition than at week −1. The concentration of vitamin B_12_ decreased significantly after calving and stayed stable afterwards. The concentrations of glucose, BHB, serum cholesterol, total serum proteins and globulin were only affected by the period (Table 4). The concentration of glucose decreased significantly after parturition, whereas the concentrations of BHB, cholesterol, total serum proteins and globulin increased.

### 3.4. Pearson’s Correlation between Fatty Acids and Blood Metabolites

The relationships between FA and blood metabolites are presented in Table A2. Generally, these relationships were very low with |r| < 0.55. The highest correlation coefficients (|r| ≥ 0.60) were observed only at week +3 relative to parturition between C6:0 and albumin, and C8:0 and globulin.

## 4. Discussion

The three dietary treatments fed to the dairy cows in the present study during the dry period are commonly used in commercial farms based on the feed that can be produced on these farms. The diets covered the requirements of the cows without excess. The dietary treatments were characterized by different nutritional compositions, which could influence the milk yield, milk composition and metabolism of the dairy cows. The main differences were linked to starch content, DVE and OEB values. The lactation diet was similar for the three treatments.

### 4.1. Effect of the Diet during the Dry Period on Milk Production and Composition

The results of this study showed that the composition of diets during the dry period had no effect on the milk yield and a limited effect on the milk FA profile. Few studies had observed the effect of the dry-period diet composition on milk production and composition. According to the results of Kokkonen et al. [31], the concentrate level in the diet given during the dry period could not affect milk yield during early and mid-lactation. Holcomb et al. [32] did not show an effect of the forage percentage in the diet during the dry period on the milk yield. The nature of forage [33] and concentrate [34] used in the diet during the dry period had no effect on the milk yield. However, in the first week of lactation, Litherland et al. [33] observed that the dairy cows produced more milk when they received orchardgrass rather than wheat straw. Many studies did not demonstrate an effect of the dietary energy level during the dry period on the milk yield [12,35], whereas, Gruber et al. [17] observed an increase in the milk yield when the energy and nutrient supply of the diet during the dry period was above 75%. Jolicoeur et al. [36] demonstrated that different management during the dry period had no effect on the milk yield. In our study, we expect that the diet during lactation can mitigate the effect of the dry-period diet on the milk traits. This observation is in agreement with the results of Dann et al. [35].

The C18:2n-6 concentration was significantly higher in the milk collected at week +3 relative to parturition for the cows receiving the MIXED treatment than those receiving the CORN treatment. For the same period, the C18:3n-3 concentration was significantly higher when the cows received the MIXED treatment compared to the CONC treatment. In accordance with our results, Morel et al. [34] observed an impact of the diet given during the dry period on the C18:2n-6 concentration, but only during the first week of lactation. During the dry period, this FA was stored in the adipose tissue and appeared to be mobilized directly during lactation. During early lactation, the milk fat was affected not only by the diet during lactation but also by the composition of the adipose tissue mobilized [37]. As observed by Conner et al. [38] in rabbits, the FA were mobilized selectively and proportionally to the degree of unsaturation and the chain length. That could explain the significant interaction observed in the present study between the dietary treatment and the week of lactation for the C18:2n-6. Furthermore, the C18:2 in the milk can be from C18:1, thanks to the action of desaturase. In the colostrum, Mann et al. [39] showed that the dry-period diet energy level had an effect on the FA profile. The FA de novo concentration increased when the dairy cows received a higher energy diet. However, in this study, the milk FA profile was weakly affected by the diet intake during the dry period. This result needs further confirmation. The results of the present study show that farmers can use different diets during the dry period without negatively affecting milk production and composition during early lactation.

### 4.2. Effect of the Diet during the Dry Period on the BCS and Serum Metabolites

The decrease in the BCS and the concomitant increase in the serum NEFA and BHB after calving observed in our trial showed that the dairy cows had an NEB in early lactation. This situation is described by other authors [8]. In accordance with our results, many studies did not observe a significant effect of the diet during the dry period on the BCS in lactation [33,40]. However, the diet could have an effect on the BCS during the dry period, according to the energy level of the diet [35]. In our study, the dietary treatment had an impact on the serum NEFA concentration. It is indicated that the NEFA concentration in the blood should not be higher than 0.4 mmol/L for dry cows and the BHB concentration in the blood should not be higher than 1.2 mmol/L for cows in early lactation [41]. In the present study, the BHB mean concentrations were higher than the recommendations, at weeks +2 and +3 in the CONC treatment (Table A1). The BHB concentration in this latter group is known to be a sign of clinical ketosis. However, in our trial, it induced no clinical symptoms and had no impact on milk yield According to the present results, NEFA concentrations increased significantly after calving only in the CORN treatment. Many studies observed a significant effect of the dry-period diet on the NEFA concentration [32,42]. In our study, the higher NEFA concentration at week +1 relative to parturition in the CORN treatment could be explained by a higher mobilization of fat because the dairy cows had a high BCS during this period. The high NEFA concentrations observed in the CORN treatment at week +1 could be an indicator of hepatic lipidosis (Table A1) [43] Furthermore, dairy cows have a lipidosis risk if the NEFA/cholesterol ratio is above 0.2 [43]. The cows in the CORN treatment receiving a higher amount of starch were the only ones to have this risk, especially at week +1. According to Drackley et al. [44], a high-starch diet during the dry period could increase the proportion of internal fat, which is more conductive to mobilization and can increase the risk of hepatic lipidosis. The starch from maize, which is more lipogenic, could lead more frequently to fat cow syndrome. In our trial, higher fat mobilization had no effect on the concentrations of BHB and glucose or on the milk yield. The lack of dry treatment effect observed on the BHB is in agreement with the results of different studies [12,45]. However, Kokkonen et al. [31] showed an increase in the BHB concentration in early lactation when the concentrate level of the diet had been increased during the dry period. Dann et al. [35] also showed that an energy restriction in the far-off dry period treatment could decrease the BHB concentration. In our study, the dairy cows can have a BHB concentration above 1.2 mmol/L, considered as subclinical ketosis [46]. However, it should be noticed that NEFA concentrations cannot be used to explain the BHB concentration. In this study, the correlation between the NEFA and BHB metabolites was very poor (r = −0.14), considering the dry and lactation periods. Contrary to the results of different studies, the increase in NEFA and BHB did not seem to affect the milk production [46,47] and the health [5] of the dairy cows. Our results showed that the serum BHB concentration, especially in the dry cows, was not a good indicator of metabolic stress.

After calving, the serum glucose decreased because of the consumption by the mammary gland (85% of the total glucose in the first three weeks without insulin dependence) and, because of the modification in the liver, induced an increase in the NEFA and BHB in the blood [48,49]. The variations in the glucose and cholesterol observed over time in our study were in accordance with the results of previous studies [45,49]. Cholesterol is the principal lipid molecule exported by the liver in lipoproteins in ruminants and is linked to the capacity of the liver to export lipids [42]. Cholesterol concentration with albumin can give information on the activity of the liver and the pro- or anti-inflammatory state of the cows [50]. In accordance with our results, the serum glucose [32,51] and cholesterol [35] were not affected by the diet during the dry period. The recommended cholesterol thresholds are 75 and 100 mg/dL for transition and early lactation, respectively. This last level was not reached in the three treatments at week +1 relative to parturition. Our results demonstrated that the increase in serum cholesterol was nevertheless dependent on the dietary treatment after calving. To our knowledge, the effect of diet on the serum cholesterol variation, during the dry period, was not studied. According to Weber et al. [49], the interaction between the dry period duration and the time had a significant effect on the serum cholesterol concentration.

Protein metabolism can be evaluated by the determination of albumin, total serum proteins, and urea in the blood. Total serum proteins and albumin concentrations can give an estimation of the globulin concentration. A high total serum protein concentration with a low albumin concentration can indicate a change in protein synthesis during an inflammatory process. In our study, albumin and total serum proteins were not influenced by diet. Bjerre-Harpøth et al. [6] observed that the dietary energy level during the dry period affected the serum albumin concentration in early lactation. The variation of the serum protein in their study agreed with our results. Contrary to Bjerre-Harpøth et al. [6], we did not observe an increase in serum albumin during the lactation of the dairy cows. According to Moorby et al. [8], the levels of two factors affected the plasma urea N concentration: feed intake and dietary protein content. Urea could also be linked to the synchronization and ratio of energy and proteins in the rumen. In our study, we observed that the serum urea could be affected by the dietary treatment given during the dry period. Moorby et al. [8] also observed an effect of the diet during the dry period on the serum urea concentration. In the present study, in contrast to the results of Moorby et al. [8], the CP level does not seem to explain the variation of the serum urea, but is consistent with the DVE level. In early lactation, we observed an increase in the serum urea concentration, probably due to a higher content of the diet in CP and DVE. We observed that the serum urea was significantly lower in the MIXED treatment than in the CONC treatment. According to the results of Mcnamara et al. [52], a low serum urea concentration could be explained by a greater capture of N in the rumen. It seemed that the capture of N was more efficient in the MIXED treatments than in the CONC treatment. The higher urea concentration observed suggests a waste of nitrogen.

The serum vitamin B_12_ is synthesized in the rumen and could provide information on rumen function. In our study, the serum vitamin B_12_ concentration of the dairy cows varied according to their diet during the dry period. It would seem that the impact of the dry-period diet on the serum vitamin B_12_ had never been studied before. Higher concentrations were observed in the MIXED treatment compared to the CONC treatment. It is also well documented that the milk vitamin B_12_ concentration could be affected by the diet during lactation [53,54]. These results show that the diet during the dry period could affect the serum vitamin B_12_. The composition of the diet and the physiological stage of the cow could affect the bacteria population of the rumen and the capacity to synthesize or use the vitamin B_12_. In the present study, the serum vitamin B_12_ decrease observed after parturition is in accordance with the results of Girard and Matte [55], which indicated that the serum vitamin B_12_ concentration varied over time. In early lactation, the vitamin B_12_ concentration was lower because of a high demand for this vitamin in the production of colostrum and milk [56] and in propionate utilization by the liver using a vitamin B_12_ dependent pathway [57]. In early lactation, as observed in our study, the serum vitamin B_12_ concentration stayed low and constant [58]. In the CONC and CORN treatments, the decrease was more severe. The MIXED diet provided starch from corn, cellulose, hemicellulose, pectin and soluble proteins from grass silage, offering a pattern of fermentation favorable for the synthesis of different elements including vitamin B. By contrast, the CONC treatment was the richest in starch. By contrast, the CONC diet contained cellulose from straw and other nutrients came from concentrate, inducing a faster fermentation profile and an increase in volatile fatty acids probably linked to concentrate intake. The CORN diet was the richest in starch and presented a negative OEB value, indicating that there is a lack of available N in the rumen. This situation can be detrimental to some categories of microbes and could lead to sub optimal fermentation. In the two latter diets (i.e., CORN and CONC), the nitrogen source was mainly storage proteins, which are less degradable in the rumen than the intracellular proteins forms included in MIXED treatment. The CORN and CONC treatments seemed to be less efficient than MIXED diet for rumen fermentation. Furthermore, the transition to a lactation diet partially composed of these silages is easier, as shown by the higher vitamin B_12_ content in the serum. According to our results, the more appropriate dry-period diet seems to be MIXED treatment.

Our study shows that, in early lactation in dairy cows, the milk production and composition is less affected by the dietary treatment than the metabolism of the cows.

## 5. Conclusions

The originality of this study was to simultaneously observe the effect of different dietary treatments during the dry period on the metabolism and milk performance of dairy cows. The diets were calculated to cover the nutrient requirements of the dairy cows without excess and had different characteristics, mainly related to the composition of starch and digestible proteins. According to our results, the diet had a more pronounced effect on the metabolism of the dairy cows (without an impact on health) than on their milk production and composition. Milk production was similar in the three treatments considered. The MIXED treatment, which is more popular on commercial farms, shows good milk production with a better composition profile and appropriate blood parameters. It appears that this diet more adequately prepares dairy cows for lactation, with the advantage of providing a smooth transition to the lactation diet. In the CONC treatment, there were signs of some difficulties with metabolism and rumen function adaptation. However, alternative diets could be used as a CONC treatment in case of a lack of forage. CORN treatment seems to be a good alternative to prepare cows for lactation, even if some signs of high fat mobilization, risk of liver lipidosis and effect of rumen function were documented. This study shows that it is possible to use different dry-period diets without having a negative effect on the milk production and FA profile in early lactation, if diets are well balanced. Further study is warranted to confirm the present results with an increased number of animals per treatment or to compare the effect of the present dietary treatments and other diets on the metabolism and the production of dairy cows during lactation.

## Figures and Tables

**Table 1:** Average ingredient and nutrient composition of the diets offered during the dry period and the lactation.

Item	Dietary Treatment ^1^			Lactation Ration (without Production Concentrate)
	CONC	CORN	MIXED	
Ingredient intake, g/kg of DM				
Corn silage	―	532	447	333
Grass silage	―	―	457	424
Alfalfa silage	―	―	―	100
Wheat straw	494	329	83	―
Barley	―	―	―	63
Lactodry ^2^	506	―	―	―
Synchromix P2 ^3^	―	126	―	77
Mineral and vitamin complement	―	13	13	3
Nutrient composition, g/kg of DM				
DM, g/kg of product	866	443	363	397
CP	123	118	121	164
Crude fat	31	28	32	35
NDF	584	479	426	332
ADF	346	298	258	212
Starch	103	175	142	188
Sugars	22	12	22	34
DVE	62	66	52	81
OEB	8	−13	10	15
NE, MJ/kg of DM	5.1	5.3	5.7	6.2
Total intake per cow, kg DM/day	10.8	10.4	10.5	20.4

DM: dry matter. CP: crude protein. NDF: neutral detergent fiber. ADF: acid detergent fiber. DVE: Intestinal digestible protein [20]. OEB: Degraded protein balance [20]. NE: Net energy for lactation calculated with the Dutch net energy evaluation (VEM) system [21]. ^1^ Dairy cows were assigned to a treatment group during the dry period: CONC: diet based on concentrate meal and straw; CORN: diet based on corn silage; MIXED: diet based on corn and grass silages. ^2^ Lactodry: concentrate (Lactodry, Quartes, Deinze, Belgium) contained (g/kg of DM): DM = 880; CP = 210; starch = 204; sugar = 43; DVE = 114; crude fiber = 119; crude fat = 51; OEB = 45; NE = 7.3 MJ/ kg of DM. ^3^ Synchromix P2: concentrate (Synchromix P2, Quartes, Deinze, Belgium) contained (g/kg of DM): DM = 880; CP = 483; starch = 65; sugar = 88; DVE = 318; OEB = 63; crude fiber = 60; crude fat = 28; NE = 7.7 MJ/ kg of DM.

**Table 2:** Description of the groups of cows which have received three dietary treatments given during the dry period.

Item	Dietary Treatment ^1^						*p*
	CONC (n = 11)		CORN (n = 11)		MIXED (n = 10)		
	Mean	SD	Mean	SD	Mean	SD	
Dry period duration (day)	50.9	5.6	50.3	14.2	49.0	8.0	0.91
Number of lactations	2.6	1.3	2.1	0.9	2.7	1.6	0.49
Pic of lactating (kg)	40.2	7.8	42.2	9.1	43.6	7.9	0.64
Milk yield (kg/day)	32.5	6.0	33.2	6.4	34.7	7.0	0.74

n: number of cows. SD: standard deviation. ^1^ Dairy cows were assigned to a treatment group during the dry period: CONC = diet based on concentrate meal and straw; CORN = diet based on corn silage; and MIXED = diet based on corn and grass silages. n = number of animals per treatment.

**Table 3:** Effects of the three dietary treatments given during the dry period on the milk yield and the milk fatty acids (FA) profile.

Item	Dietary Treatment (T) ^1^						Period (W) ^2^						*p*		
	CONC		CORN		MIXED		W1		W2		W3				
	Emmean	SE	Emmean	SE	Emmean	SE	Emmean	SE	Emmean	SE	Emmean	SE	T	W	T × W
Milk yield (kg/d)	29.08	2.28	30.10	2.28	33.27	2.40	26.22a	1.39	32.38b	1.39	33.85b	1.40	0.43	<0.001	
FA (g/100 g of total FA)															
C4:0	2.97	0.05	3.05	0.05	3.03	0.05	3.02	0.04	2.98	0.04	3.06	0.05	0.50	0.55	
C6:0	1.94	0.05	1.97	0.05	1.97	0.05	1.96	0.04	1.98	0.04	1.95	0.05	0.83	0.84	
C8:0	1.21	0.04	1.23	0.04	1.25	0.04	1.21	0.04	1.26	0.04	1.22	0.04	0.80	0.66	
C10:0	2.95	0.13	3.02	0.14	3.05	0.14	2.90	0.12	3.04	0.12	3.08	0.13	0.87	0.54	
C12:0	3.45	0.16	3.57	0.17	3.56	0.17	3.40	0.16	3.57	0.16	3.61	0.17	0.83	0.62	
C14:0	11.59	0.34	11.58	0.37	12.19	0.37	11.61	0.35	11.80	0.35	11.96	0.39	0.41	0.80	
C14:1	0.84	0.04	0.83	0.05	0.91	0.05	0.84	0.04	0.86	0.04	0.89	0.05	0.41	0.80	
C16:0	31.53	0.92	31.47	0.97	31.58	0.99	31.91	0.84	31.18	0.84	31.50	0.93	1	0.81	
cis-9C16:1	1.74	0.06	1.63	0.07	1.67	0.07	1.70	0.06	1.65	0.06	1.69	0.07	0.49	0.86	
C17:0	0.77	0.01	0.75	0.01	0.76	0.01	0.76	0.01	0.75	0.01	0.77	0.01	0.47	0.36	0.06
C18:0	10.54	0.38	11.26	0.41	10.41	0.41	10.79	0.39	10.76	0.39	10.67	0.43	0.29	0.80	
cis-9C18:1	19.59	1.01	19.09	1.07	18.77	1.09	19.57	1.03	18.81	1.03	19.08	1.12	0.86	0.87	
C18:2n-6	2.03	0.08	1.78	0.08	2.03	0.09	1.87	0.08	1.94	0.08	2.02	0.09	0.06	0.43	0.02
C18:3n-3	0.51	0.02	0.55	0.02	0.54	0.02	0.51	0.02	0.53	0.02	0.56	0.02	0.50	0.23	0.005
Total unsaturated FA	28.31	0.99	29.70	1.09	28.79	1.09	30.12	1.05	29.12	1.03	27.57	1.09	0.64	0.24	
SCFA	9.57	0.26	9.77	0.27	9.88	0.28	9.65	0.22	9.72	0.22	9.85	0.24	0.71	0.78	
MCFA	52.85	1.29	51.95	1.38	53.65	1.39	53.04	1.26	52.30	1.26	53.12	1.39	0.69	0.88	
LCFA	34.90	1.48	38.10	1.60	36.64	1.64	38.16	1.57	36.77	1.51	34.72	1.64	0.34	0.32	

Emmean: estimated marginal means. LCFA, long chain fatty acids. MCFA: mean chain fatty acids. SCFA: short chain fatty acids. SE: standard error. ^1^ Dairy cows were assigned to a treatment group during the dry period: CONC = diet based on concentrate meal and straw; CORN = diet based on corn silage; and MIXED = diet based on corn and grass silages. ^2^ Period: W1, W2 and W3 corresponding to weeks +1, +2 and +3 relative to calving, respectively.

**Table 4:** Effects of the three dietary treatments given during the dry period on the body condition score (BCS) and blood metabolites.

Item	Dietary Treatment (T) ^1^						Period (W) ^2^								*p*		
	CONC		CORN		MIXED		W − 1		W1		W2		W3				
	Emmean	SE	Emmean	SE	Emmean	SE	Emmean	SE	Emmean	SE	Emmean	SE	Emmean	SE	T	W	T × W
BCS	2.44	0.07	2.60	0.07	2.66	0.07	2.74 ^c^	0.05	2.60 ^b^	0.05	2.51 ^a,b^	0.05	2.43 ^a^	0.06	0.06	<0.001	
Blood metabolites																	
Glucose (mg/dL)	38.58	1.89	39.10	1.83	37.47	1.98	43.55 ^b^	1.71	36.99 ^a^	1.69	34.48 ^a^	1.77	38.51 ^a,b^	1.74	0.82	<0.001	
BHB (mmol/L)	1.07	0.11	0.92	0.11	0.95	0.11	0.71 ^a^	0.10	0.93 ^a,b^	0.09	1.15 ^b^	0.10	1.13 ^b^	0.10	0.58	<0.001	
Cholesterol (mg/dL)	101.90	5.19	99.07	5.07	105.86	5.50	79.37 ^a^	3.97	87.39 ^a^	3.93	109.95 ^b^	4.07	132.40 ^c^	4.07	0.67	<0.001	
NEFA (mmol/L)	0.21 ^a^	0.04	0.38 ^b^	0.04	0.33 ^a,b^	0.05	0.28 ^a^	0.04	0.42 ^b^	0.04	0.27 ^a^	0.04	0.26 ^a^	0.04	0.04	0.006	0.05
NEFA/Cholesterol ratio	0.09	0.02	0.16	0.02	0.13	0.02	0.14 ^a,b^	0.02	0.20 ^b^	0.02	0.10 ^a^	0.02	0.07 ^a^	0.02	0.08	<0.001	0.03
Urea (mg/dL)	27.07 ^b^	0.97	25.24 ^a,b^	0.97	22.05 ^a^	1.06	21.93 ^a^	1.03	26.15 ^b^	1.02	25.22 ^a,b^	1.07	25.85 ^b^	1.09	0.006	0.01	
TSP (g/L)	64.77	1.05	66.56	1.02	66.83	1.11	62.36 ^a^	0.79	65.10 ^b^	0.78	67.75 ^c^	0.81	69.00 ^c^	0.81	0.34	<0.001	
Albumin (g/L)	32.78	0.90	33.07	0.87	33.35	0.95	33.38	0.61	33.05	0.61	32.89	0.62	32.96	0.63	0.91	0.81	0.03
Globulin (g/L)	31.96	1.32	33.55	1.28	33.41	1.39	29.5 ^a^	0.95	32.05 ^b^	0.95	34.84 ^c^	0.97	35.97 ^c^	0.97	0.65	<0.001	
Vitamin B_12_ (ng/L)	156.28 ^a^	12.06	165.14 ^a,b^	11.23	201.13 ^b^	12.17	200.13 ^b^	9.02	168.33 ^a^	8.96	168.74 ^a^	9.26	159.54 ^a^	9.31	0.04	<0.001	

BHB: β-hydroxybutyrate. Emmean: estimated marginal means. NEFA: non-esterified fatty acids. SE: standard error. TSP: total serum proteins. ^1^ Dairy cows were assigned to a treatment group during the dry period: CONC = diet based on concentrate meal and straw; CORN = diet based on corn silage; and MIXED = diet based on corn and grass silages. ^2^ Period: W−1, W1, W2 and W3 corresponding to weeks −1, +1, +2 and +3 relative to calving, respectively. Values followed by different letters (a, b, c) are significantly different (*p* ≤ 0.05).

## Appendix A

**Table A1:** Variation of blood metabolites according to the dietary treatment.

Blood Metabolites	Dietary Treatment ^1^																							
	CONC								CORN								MIXED							
	W−1		W1		W2		W3		W−1		W1		W2		W3		W−1		W1		W2		W3	
	Emmean	SE	Emmean	SE	Emmean	SE	Emmean	SE	Emmean	SE	Emmean	SE	Emmean	SE	Emmean	SE	Emmean	SE	Emmean	SE	Emmean	SE	Emmean	SE
Glucose (mg/dL)	43.74	2.32	37.18	2.27	34.67	2.37	38.71	2.29	44.27	2.28	37.71	2.22	35.20	2.31	39.24	2.26	42.63	2.33	36.07	2.38	33.56	2.38	37.60	2.45
BHB (mmol/L)	0.80	0.13	1.03	0.13	1.25	0.13	1.22	0.13	0.65	0.13	0.87	0.13	1.10	0.13	1.07	0.13	0.67	0.13	0.90	0.14	1.12	0.13	1.09	0.14
Cholesterol (mg/dL)	78.99	5.81	87.02	5.73	109.58	5.89	132.03	5.84	76.16	5.74	84.19	5.64	106.75	5.78	129.19	5.70	82.95	5.99	90.97	6.09	113.53	6.07	135.98	6.21
NEFA (mmol/L)	0.26	0.07	0.24	0.07	0.20	0.07	0.15	0.07	0.22	0.07	0.56	0.07	0.33	0.07	0.41	0.07	0.36	0.07	0.46	0.08	0.28	0.07	0.22	0.08
NEFA/Cholesterol ratio	0.15	0.03	0.11	0.03	0.07	0.04	0.04	0.03	0.11	0.04	0.30	0.03	0.12	0.04	0.12	0.03	0.17	0.03	0.19	0.04	0.10	0.04	0.06	0.04
Urea (mg/dL)	24.21	1.30	28.44	1.27	27.50	1.34	28.14	1.32	22.38	1.32	26.60	1.27	25.67	1.34	26.31	1.32	19.19	1.33	23.41	1.37	22.48	1.36	23.12	1.45
TSP (g/L)	61.08	1.16	63.82	1.15	66.46	1.18	67.72	1.17	62.87	1.15	65.61	1.13	68.25	1.15	69.51	1.14	63.14	1.20	65.87	1.22	68.52	1.21	69.78	1.24
Albumin (g/L)	31.46	1.05	33.00	1.03	33.20	1.08	33.47	1.05	34.57	1.06	32.90	1.01	32.13	1.06	32.69	1.03	34.10	1.08	33.24	1.14	33.33	1.11	32.74	1.18
Globulin (g/L)	28.01	1.44	31.03	1.42	33.83	1.46	34.95	1.44	29.62	1.41	32.62	1.39	35.42	1.42	36.55	1.40	29.48	1.49	32.48	1.51	35.28	1.50	36.40	1.53
Vitamin B_12_ (ng/L)	183.04	13.70	150.85	13.52	151.29	13.91	141.44	13.77	190.64	13.02	158.46	12.78	158.89	13.12	149.04	12.92	227.59	13.58	195.40	13.80	195.84	13.80	185.99	14.09

BHB: β-hydroxybutyrate. Emmean: estimated marginal means. NEFA: non-esterified fatty acids. SE: standard error. TSP: total serum proteins. W−1, W1, W2 and W3 corresponding to weeks −1, +1, +2 and +3 relative to parturition, respectively. ^1^ Dairy cows were assigned to a treatment group during the dry period: CONC = diet based on concentrate meal and straw; CORN = diet based on corn silage; and MIXED = diet based on corn and grass silages.

## Appendix B

Table A2 Pearson’s correlations between fatty acids (FA) and blood metabolites at different periods after parturition: weeks +1 (A), +2 (B) and +3 (C), respectively.

**Table:** **A. Pearson’s correlations at week +1 relative to parturition.**

	Glucose	BHB	Cholesterol	NEFA	NEFA/Cholesterol Ratio	Urea	TSP	Albumin	Globulin	Vitamin B_12_
C4:0	−0.11	0.03	−0.02	−0.02	0.02	−0.02	−0.19	−0.21	−0.01	−0.07
C6:0	−0.09	0.24	−0.04	0.11	0.06	−0.13	−0.12	−0.05	−0.06	−0.05
C8:0	0.01	−0.02	−0.01	−0.002	−0.04	−0.31	−0.08	−0.13	0.03	−0.14
C10:0	0.01	0.09	−0.20	0.05	0.05	−0.18	−0.15	−0.09	−0.06	−0.04
C12:0	0.05	0.05	−0.20	0.03	0.03	−0.22	−0.09	−0.03	−0.05	−0.01
C14:0	0.04	0.11	−0.23	0.07	0.07	−0.12	−0.05	0.03	−0.06	−0.003
C14:1	0.17	0.09	−0.18	0.28	0.28	0.05	0.0002	0.35	−0.24	−0.03
C16:0	−0.0	0.09	−0.14	0.27	0.27	−0.12	0.01	0.13	−0.07	−0.03
cis9C16:1	−0.07	0.17	0.06	0.10	0.08	0.32	0.14	0.46	−0.20	−0.03
C17:0	−0.09	0.29	−0.26	0.23	0.25	0.20	−0.17	0.04	−0.16	0.27
C18:0	−0.03	−0.28	0.14	−0.22	−0.18	−0.06	0.03	−0.20	0.16	−0.08
cis9C18:1	−0.005	−0.10	0.15	−0.01	−0.01	0.17	0.07	0.10	−0.01	−0.005
C18:2n-6	−0.03	0.17	−0.22	−0.18	−0.15	0.08	−0.11	0.06	−0.13	0.009
C18:3n-3	0.01	−0.05	0.04	−0.15	−0.16	−0.02	−0.18	−0.33	0.07	−0.01
Total unsaturated FA	−0.06	−0.16	0.24	−0.01	−0.03	0.06	−0.07	−0.01	−0.05	−0.07
SCFA	−0.06	0.21	−0.25	0.01	0.04	−0.10	−0.24	−0.16	−0.08	0.01
MCFA	−0.01	0.21	−0.24	0.19	0.21	−0.03	−0.02	0.14	−0.11	0.07
LCFA	0.07	−0.28	0.36	−0.02	−0.07	−0.18	0.24	0.10	0.12	0.01

**Table:** **B. Pearson’s correlations at week +2 relative to parturition.**

	Glucose	BHB	Cholesterol	NEFA	NEFA/Cholesterol Ratio	Urea	TSP	Albumin	Globulin	Vitamin B_12_
C4:0	−0.51	0.33	−0.13	−0.003	0.01	0.17	0.07	0.19	−0.07	0.20
C6:0	−0.002	−0.09	0.25	−0.37	−0.48	0.12	−0.31	−0.07	−0.22	−0.10
C8:0	0.02	−0.11	0.31	−0.33	−0.47	0.09	−0.15	0.08	−0.18	−0.21
C10:0	0.14	−0.24	0.35	−0.39	−0.54	0.01	−0.23	−0.03	−0.18	−0.24
C12:0	0.15	−0.27	0.36	−0.41	−0.57	−0.03	−0.29	−0.07	−0.20	−0.25
C14:0	0.14	−0.20	0.28	−0.38	−0.50	−0.07	−0.31	−0.05	−0.23	−0.26
C14:1	0.16	−0.13	0.18	−0.05	−0.13	−0.17	−0.14	−0.17	−0.005	−0.02
C16:0	−0.09	0.12	−0.01	−0.20	−0.15	−0.09	−0.53	−0.19	−0.32	0.08
cis9C16:1	0.16	0.003	−0.16	0.25	0.34	−0.09	0.08	−0.005	0.07	−0.04
C17:0	0.46	−0.24	0.14	−0.09	−0.14	0.07	−0.03	−0.22	0.13	−0.06
C18:0	−0.30	0.25	−0.16	0.27	0.31	0.25	0.26	0.21	0.07	0.08
cis9C18:1	−0.08	0.13	−0.29	0.40	0.50	−0.02	0.38	0.004	0.32	0.20
C18:2n-6	0.40	−0.22	0.31	−0.25	−0.35	0.04	0.02	0.25	−0.16	−0.45
C18:3n-3	0.09	−0.11	0.13	−0.04	−0.12	0.26	0.14	0.01	0.11	−0.04
Total unsaturated FA	−0.11	0.16	−0.22	0.41	0.49	0.07	0.45	0.11	0.30	0.13
SCFA	0.01	−0.14	0.22	−0.41	−0.51	0.02	−0.25	−0.14	−0.11	−0.08
MCFA	0.04	−0.04	0.15	−0.33	−0.35	−0.11	−0.54	−0.20	−0.32	−0.04
LCFA	−0.23	0.22	−0.26	0.39	0.48	0.10	0.40	0.10	0.27	0.19

**Table:** **C. Pearson’s correlations at week +3 relative to parturition.**

	Glucose	BHB	Cholesterol	NEFA	NEFA/Cholesterol Ratio	Urea	TSP	Albumin	Globulin	Vitamin B_12_
C4:0	0.06	0.06	−0.29	−0.04	0.10	0.05	0.29	−0.15	0.29	0.12
C6:0	−0.27	0.15	−0.55	−0.21	−0.07	−0.29	0.10	−0.63	0.47	0.367
C8:0	−0.24	0.05	−0.59	−0.06	−0.000	−0.34	0.43	−0.49	0.60	0.19
C10:0	0.07	−0.05	−0.51	−0.20	−0.16	−0.15	0.44	−0.37	0.54	0.15
C12:0	0.02	0.03	−0.48	−0.17	−0.14	−0.18	0.37	−0.42	0.51	0.21
C14:0	0.01	0.06	−0.46	−0.10	−0.09	−0.13	0.43	−0.30	0.48	0.22
C14:1	0.17	0.13	−0.31	−0.29	−0.13	0.01	−0.03	−0.26	0.14	0.40
C16:0	0.04	0.25	−0.38	−0.30	−0.11	−0.06	−0.03	−0.54	0.32	0.14
cis9C16:1	0.21	0.06	0.52	−0.12	−0.16	0.44	−0.52	0.44	−0.63	−0.04
C17:0	0.20	−0.21	0.02	−0.46	−0.51	0.13	−0.08	0.001	−0.05	0.19
C18:0	−0.17	−0.08	0.13	0.32	0.28	−0.04	0.13	0.21	−0.05	−0.34
cis9C18:1	0.11	−0.06	0.47	0.19	0.10	0.31	−0.19	0.53	−0.46	−0.21
C18:2n-6	0.08	−0.30	0.23	0.11	−0.10	0.15	0.14	0.48	−0.22	−0.30
C18:3n-3	−0.13	−0.29	−0.31	0.25	0.14	−0.29	0.57	−0.02	0.39	0.03
Total unsaturated FA	−0.17	−0.09	0.28	0.43	0.30	0.01	0.003	0.38	−0.24	−0.13
SCFA	0.04	−0.03	−0.48	−0.24	−0.16	−0.14	0.33	−0.40	0.47	0.24
MCFA	0.01	0.17	−0.39	−0.27	−0.14	−0.13	0.02	−0.53	0.35	0.23
LCFA	−0.26	−0.02	0.25	0.42	0.33	−0.04	−0.04	0.26	−0.19	−0.14

BHB: β-hydroxybutyrate. LCFA, long chain fatty acids. MCFA: mean chain fatty acids. NEFA: non-esterified fatty acids. SCFA: short chain fatty acids. TSP: total serum proteins.
